# SARS-CoV-2 cellular and humoral responses in vaccine-naive individuals during the first two waves of COVID-19 infections in the southern region of The Netherlands: a cross-sectional population-based study

**DOI:** 10.1128/spectrum.00126-24

**Published:** 2024-04-30

**Authors:** D. A. T. Hanssen, K. Arts, W. H. V. Nix, N. N. B. Sweelssen, T. T. J. Welbers, C. de Theije, L. Wieten, D. M. E. Pagen, S. Brinkhues, J. Penders, N. H. T. M. Dukers-Muijrers, C. J. P. A. Hoebe, P. H. M. Savelkoul, I. H. M. van Loo

**Affiliations:** 1 Department of Medical Microbiology, Infectious Diseases and Infection Prevention, Maastricht University Medical Center, Maastricht, The Netherlands; 2 Care and Public Health Research Institute (CAPHRI), Maastricht University, Maastricht, The Netherlands; 3 BioBank Maastricht UMC+, Maastricht University Medical Center, Maastricht, The Netherlands; 4 Department of Transplantation Immunology, Maastricht University Medical Center, Maastricht, The Netherlands; 5 Department of Sexual Health, Infectious Diseases and Environmental Health, Living Lab Public Health, Public Health Service (GGD) South Limburg, Heerlen, The Netherlands; 6 Department of Social Medicine, Maastricht University, Maastricht, The Netherlands; 7 Department of Knowledge and Innovation, Public Health Service (GGD) South Limburg, Heerlen, The Netherlands; 8 Department of Medical Microbiology, School of Nutrition and Translational Research in Metabolism (NUTRIM), Maastricht University Medical Center, Maastricht, The Netherlands; 9 Department of Health Promotion, Maastricht University, Maastricht, The Netherlands; National Institute of Allergy and Infectious Diseases, Baltimore, Maryland, USA

**Keywords:** SARS-CoV-2, cellular immunity, peripheral blood mononuclear cell, antibody response, ELISpot, IFN_γ _response

## Abstract

With the emergence of highly transmissible variants of concern, severe acute respiratory syndrome coronavirus 2 (SARS-CoV-2) still poses a global threat of coronavirus disease 2019 (COVID-19) resurgence. Cellular responses to novel variants are more robustly maintained than humoral responses, and therefore, cellular responses are of interest in assessing immune protection against severe disease in the population. We aimed to assess cellular responses to SARS-CoV-2 at the population level. IFN_γ_ (interferon _γ_) responses to wild-type SARS-CoV-2 were analyzed using an ELISpot assay in vaccine-naive individuals with different humoral responses: Ig (IgM and/or IgG) seronegative (*n* = 90) and seropositive (*n* = 181) with low (<300 U/mL) or high (≥300 U/mL) humoral responses to the spike receptor binding domain (anti-S-RBD). Among the seropositive participants, 71.3% (129/181) were IFN_γ_ ELISpot positive, compared to 15.6% (14/90) among the seronegative participants. Common COVID-19 symptoms such as fever and ageusia were associated with IFN_γ_ ELISpot positivity in seropositive participants, whereas no participant characteristics were associated with IFN_γ_ ELISpot positivity in seronegative participants. Fever and/or dyspnea and anti-S-RBD levels were associated with higher IFN_γ_ responses. Symptoms of more severe disease and higher anti-S-RBD responses were associated with higher IFN_γ_ responses. A significant proportion (15.6%) of seronegative participants had a positive IFN_γ_ ELISpot. Assessment of cellular responses may improve estimates of the immune response to SARS-CoV-2 in the general population.

**IMPORTANCE:**

Data on adaptive cellular immunity are of interest to define immune protection against severe acute respiratory syndrome coronavirus 2 in a population, which is important for decision-making on booster-vaccination strategies. This study provides data on associations between participant characteristics and cellular immune responses in vaccine-naive individuals with different humoral responses.

## INTRODUCTION

Adaptive immunity to coronavirus infection involves an interaction between humoral and cellular immune responses. In general, neutralizing antibodies mainly protect against contracting infection, whereas cellular immune responses mainly play an important role in fighting the virus once the infection has occurred. Early induction of the cellular immune response is associated with mild disease and rapid viral clearance, helping to prevent hospitalization and death (1, 2). Individuals with inadequate or absent antibody responses can still induce cellular immune responses, and these responses have been associated with less severe disease and the ability to contain severe acute respiratory syndrome coronavirus 2 (SARS-CoV-2) infection (3). These observations highlight the importance of cellular immune responses in combating the virus when neutralizing antibody levels are suboptimal or absent.

In addition to the role of cellular immunity in containing the virus once infected, studies in animals and humans have shown that memory cellular immune responses play an important role in preventing reinfection (4, 5). While new variants of SARS-CoV-2 have demonstrated the ability to evade neutralizing antibodies (6), T-cell immunity induced by vaccination or infection with previous variants can still recognize the Omicron variant (7–9). Therefore, more data on adaptive cellular immunity are of interest to define immune protection against SARS-CoV-2 in a population.

In late 2020, a cross-sectional community-based study was conducted in residents of a southern province of the Netherlands to estimate the seroprevalence of antibodies directed against SARS-CoV-2 after the first two waves of SARS-CoV-2 infection. In addition to serum samples to investigate humoral immune responses, peripheral blood mononuclear cells (PBMCs) were collected to measure cellular immune responses against SARS-CoV-2. This study aims to elucidate the extent of cellular immune responses in participants with different humoral immune responses (seropositive vs seronegative). In addition, we aim to identify potential predictors of SARS-CoV-2-directed cellular memory immune responses.

## MATERIALS AND METHODS

### Study design

**Fig 1:**
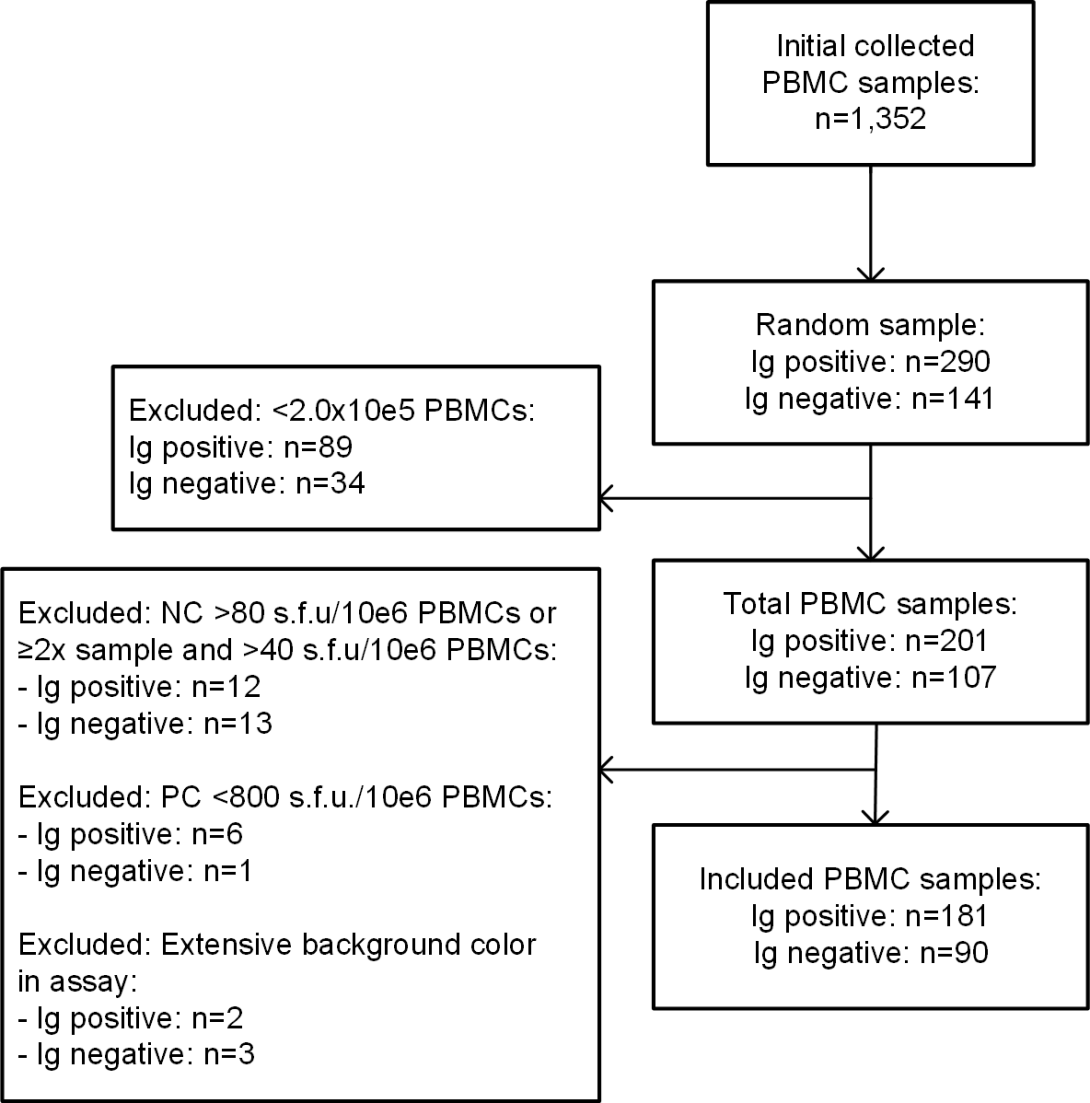
Inclusion of study population.

This study was part of a cross-sectional SARS-CoV-2 seroprevalence study involving 10,001 inhabitants of the province of Limburg, located in the southern region of the Netherlands (10). From 28 October to 30 November 2020, PBMCs were isolated from 13.5% (1,352/10,001) of randomly selected participants. The present study included PBMC samples from 290 randomly selected participants who tested seropositive with the Wantai SARS-CoV-2 Ab enzyme-linked immunosorbent assay (ELISA) (Ig; which detects IgM and/or IgG) and 141 randomly selected Ig seronegative participants. Samples were excluded if the predefined criteria for the ELISpot assay were not met, resulting in the inclusion of 181 PBMC samples from seropositive and 90 PBMC samples from seronegative participants (Fig. 1).

In addition, questionnaires were collected that included participants’ characteristics (sex and age) and experienced symptoms (10). Participants with fever, dyspnea, muscle ache, extreme fatigue, malaise, painful respiration, diarrhea, stomach ache, anosmia, and/or ageusia were considered to have coronavirus disease 2019 (COVID-19) compatible symptoms. Participants with fever or dyspnea were considered to have more severe disease. Asymptomatic participants or participants with symptoms such as cough, throat soreness, rhinorrhea, and/or headache were grouped together as these symptoms are not specific to COVID-19. In addition, participants were asked if they had ever been hospitalized for COVID-19.

To gain insight into the association between the time of infection and cellular immune responses, we categorized the onset of infection as 6–9 months prior to sample collection, corresponding to the first wave of SARS-CoV-2 infection in the Netherlands, or ≤5 months before sample collection, corresponding to the second wave of infection. Infection onset was mainly based on the date of a positive PCR (*n* = 43). At the beginning of the SARS-CoV-2 pandemic in the Netherlands, the PCR testing policy in the Netherlands was strict, including only hospitalized individuals, healthcare workers, or individuals at high risk of complicated COVID-19. From June, any individual with symptoms was eligible for PCR testing. Thus, participants with COVID-19-compatible symptoms with onset 6–9 months prior to sample collection who were not tested by PCR during this period were categorized as possible cases (*n* = 199).

### Diagnostic tests

#### Antibody assays

The Wantai SARS-CoV-2 Ab (Ig) ELISA (Beijing Wantai Biological Pharmacy Enterprise Co., Ltd, Beijing, China) was used to determine qualitative antibody responses, including IgM and IgG (Ig) (Virion/Serion Immunomat, Virion/Serion, Würzburg, Germany) (11). The Wantai SARS-CoV-2 Ab (Ig) ELISA is considered positive when the absorbance to cut-off ratio is ≥1.1, and borderline when the absorbance to cut-off ratio is ≥0.9 to <1.1. In participants with borderline or positive results, the Elecsys anti-SARS-CoV-2 S electrochemiluminescence immunoassay (Roche Diagnostics GmbH, Mannheim, Germany) was additionally performed to determine the quantitative antibody response to SARS-CoV-2 infection using the Cobas 8000 (Roche Diagnostics GmbH, Mannheim, Germany) (12). Both tests were performed according to the manufacturer’s instructions. The Elecsys anti-SARS-CoV-2 S test quantitatively detects total antibodies to the SARS-CoV-2 spike receptor binding domain (anti-S-RBD), and values ≥0.8 U/mL are considered positive. Samples with values ≥250 U/mL were retested at a 1:4 dilution using diluent buffer (Roche Diagnostics GmbH, Mannheim, Germany). The assigned units per milliliter is comparable to the binding antibody units per milliliter, the WHO International Standard for COVID-19 serological tests (13).

#### Anti-S-RBD response

The anti-S-RBD response was analyzed in two ways: quantitatively and dichotomized into negative/low-positive (<300 U/mL) or high-positive (≥300 U/mL) anti-S-RBD results to investigate whether cellular immune responses compensated for suboptimal humoral immune responses. As levels of spike-binding IgG antibody levels of 264 BAU/mL and 298 BAU/mL were associated with 80%–90% protection against symptomatic infection with the wild-type, alpha, and delta variants of SARS-CoV-2 in vaccination studies, this level was chosen as the cut-off (14).

#### PBMC isolation

PBMCs were isolated from EDTA plasma within 6 hours of sample collection and were stored at room temperature before further processing. PBMCs were isolated by centrifugation at 2,000 × *g* (relative centrifugal force) for 10 minutes at 4°C, after which the buffy coat was transferred to a Falcon tube filled with 7 mL Hanks’ balanced salt solution (HBSS). The buffy coat was then resuspended in HBSS and transferred to a SepMate-15 PBMC isolation tube (Stemcell Technologies Canada Inc., Vancouver, Canada), filled with 4 mL lymphoprep (Stemcell Technologies Canada Inc., Vancouver, Canada). The SepMate-15 PBMC isolation tube was centrifuged at 1,200 × *g* for 10 minutes at 4°C. The PBMC layer was then washed by transferring the PBMC layer to a Greiner tube filled with 10 mL of wash buffer (HBSS containing 2% fetal calf serum). The suspension was centrifuged at 300 × *g* for 8 minutes at 4°C. The cell pellet was washed again with wash buffer (HBSS containing 2% fetal calf serum). Another centrifugation step followed for 8 minutes at 300 × *g* at 4°C. The supernatant was discarded, and the PBMCs were resuspended in 3 mL of medium [50% RPMI-1640 containing L-glutamine, 40% fetal calf serum, and 10% dimethyl sulfoxide (DMSO)] and gradually frozen at −80°C in Corning CoolCell boxes and transferred to −196°C at 24 hours until further analysis.

#### ELISpot assay

Cellular responses were assessed by ELISpot using the Human IFN_γ_ ELISpot^PLUS^ kit (ALP) (Mabtech AB, Nacka Strand, Sweden). Per well, 2 × 10^5^ PBMCs were stimulated with the Peptivator SARS-CoV-2 Select peptide pool (6 nmol/peptide, Peptivator, SARS-CoV-2 Select, premium grade, Miltenyi Biotec, Bergisch Gladbach, Germany) at a final concentration of 1 µg/mL. The Peptivator SARS-CoV-2 Select peptide pool contains 88 peptides and is derived from structural proteins (S, M, N, and E) and non-structural proteins. ELISpot plates were incubated at 37°C for 18 hours. The Human IFN_γ_ ELISpot^PLUS^ assay (Mabtech AB, Nacka Strand, Sweden) was performed according to the manufacturer’s instructions.

#### Spot counting

Spots were counted using the AID iSpot, AID GmbH, Strassberg, Germany; AID ELISpot Software version 7 with the following thresholds: intensity 60–255, size 130–5,000, gradient 30–90. Responses were expressed as spot-forming counts (s.f.c.) per 10^6^ PBMCs. We determined mean background levels of 5 s.f.c. per negative well (range 0–16, standard deviation 4), and defined the lower limit of detection at 10 s.f.c./10^6^ PBMCs. The intra-assay coefficient of variability (CV) for SARS-CoV-2-naive individuals with IFN_γ_ responses between 1–10 s.f.c./10^6^ PBMCs was of 32.3%. The intra-assay CV of IFN_γ_ responses of ≥25 s.f.c./10^6^ PBMC was 10.1%. To quantify cellular responses, spots from the negative wells were subtracted from the stimulated wells. Stimulated wells were considered positive if the result of the stimulated spot was at least three times that of the spots in the negative well and at least ≥25 s.f.c./10^6^ PBMC (15). Samples were excluded if the negative well had >80 s.f.c./10^6^ PBMCs, the negative well had >2 times the number of spot-forming counts per 10^6^ PBMCs than the sample and >40 s.f.c./10^6^ PBMCs, or the positive well had <800 s.f.c./10^6^ PBMCs.

### Statistical analysis

SPSS version 26.0 was used for statistical analysis. The Pearson *χ*^2^ test was used to analyze relationships between categorical variables. Fisher’s exact test was used for expected values <5. The Mann-Whitney *U* test was used to compare IFN_γ_ responses for continuous variables with two categories. Spearman’s correlation was calculated to analyze the quantitative correlation between the anti-S-RBD and IFN_γ_ responses. A two-sided *P* value ≤0.05 was considered to be statistically significant.

## RESULTS

### Participant characteristics

The study included 87 males (32.1%) and 184 females (67.9%) with a median age of 47 years (Interquartile range (IQR) 39–61) and 43 years (IQR 33–56), respectively (Table 1). The date of a positive PCR was known for 43 participants (15.9%). Eleven participants had a positive PCR 6–9 months prior to sample collection, while 32 participants reported a positive PCR ≤5 months prior to sample collection.

**TABLE 1:** Characteristics of study participants (*n* = 271)

	Total study population (*n* = 271)	ELISpot positive (*n* = 143)	ELISpot negative (*n* = 128)	Sig. (2-sided)
Sex, *n* (%)				*P* = 0.57
Male	87 (32.1)	48 (55.2)	39 (44.8)	
Female	184 (67.9)	95 (51.6)	89 (48.4)	
Age (years), median (IQR)	44 (34–57)	48 (36–60)	43 (31–53)	*P* < 0.01^**^
Comorbidities^*b*^, *n* (%)				*P* = 0.68
No	198 (73.1)	106 (53.5)	92 (46.5)	
Yes	73 (26.9)	37 (50.7)	36 (49.3)	
Medication, *n* (%)				
Immunosuppressant	10 (3.7)	5 (50.0)	5 (50.0)	*P* = 1.00^*a*^
Chemotherapy	0 (0.0)	0 (0.0)	0 (0.0)	
Anti-infective	49 (18.1)	25 (51.0)	24 (49.0)	*P* = 0.79
Symptoms, *n* (%)				
Cough	190 (70.1)	92 (48.4)	98 (51.6)	*P* = 0.03^*^
Throat soreness	181 (66.8)	80 (44.2)	101 (55.8)	*P* < 0.001^***^
Rhinorrhea	192 (70.8)	92 (47.9)	100 (52.1)	*P* = 0.01^*^
Dyspnea	132 (48.7)	71 (53.8)	61 (46.2)	*P* = 0.74
Painful respiration	61 (22.5)	28 (45.9)	33 (54.1)	*P* = 0.22
Fever	133 (49.1)	82 (61.7)	51 (38.3)	*P* < 0.01^**^
Muscle pain	143 (52.8)	74 (51.7)	69 (48.3)	*P* = 0.72
Anosmia	119 (43.9)	81 (68.1)	38 (31.9)	*P* < 0.001^***^
Aguesia	130 (48.0)	93 (71.5)	37 (28.5)	*P* < 0.001^***^
Headache	193 (71.2)	94 (48.7)	99 (51.3)	*P* = 0.04^*^
Extreme fatigue	221 (81.5)	120 (54.3)	101 (45.7)	*P* = 0.29
General malaise	194 (71.6)	102 (52.6)	92 (47.4)	*P* = 0.92
Stomach ache	67 (24.7)	34 (50.7)	33 (49.3)	*P* = 0.70
Diarrhea	86 (31.7)	44 (51.2)	42 (48.8)	*P* = 0.72
Ig serostatus				*P* < 0.001^***^
Positive	181 (66.8)	129 (71.3)	52 (28.7)	
Negative	90 (33.2)	14 (15.6)	76 (84.4)	
Anti-S-RBD (U/mL)				
<300	151 (55.5)	101 (66.9)	50 (33.1)	
≥300	30 (11.0)	28 (93.3)	2 (6.7)	
Missing	91 (33.5)			*P* < 0.01^**^
Number of days between positive PCR and sample (*n* = 43), median (IQR)	35 (24–177)	46 (25–221)	34 (23–38)	*P* = 0.08
Period of infection				*P* = 0.08
6–9 months	210 (77.5)	118 (56.2)	92 (43.8)	
≤5 months	45 (16.6)	17 (73.8)	28 (62.2)	
Missing^*c*^	16 (5.9)			
IFN_γ_ ELISpot^PLUS^ (s.f.u./10^6^ PBMCs), median (IQR)		145 (80–310)		

^*a*^ In case of expected counts <5, Fisher’s exact test was used.

^*b*^ Comorbidities: pulmonary, cardiovascular, immune, hematologic or solid organ transplant, malignancy, liver, kidney, skin, rheumatic, neurologic.

^*c*^ Participants who could not be categorized in the first or second wave because of being asymptomatic, or reporting non-specific symptoms. Significant values are displayed in bold. *P < 0.05, **P < 0.01, and ***P < 0.001.

The majority of participants (67.5%) reported fever and/or dyspnea (183/271), while 26.9% (73/271) reported mild COVID-19 compatible symptoms. Sixteen participants (5.9%) were asymptomatic or reported non-specific symptoms.

Anti-S-RBD levels were ≥300 U/mL in 16.6% of Ig seropositive participants (30/181). Participants with anti-S-RBD levels ≥300 U/mL were significantly older [61 years (IQR 50–65)] than those participants with anti-S-RBD levels <300 U/mL [44 years (IQR 32–57)], *P* < 0.001, and were more likely to have comorbidities, *P* < 0.01.

### ELISpot responses

No differences were observed in the number of spots in the negative wells between samples from Ig seropositive and seronegative participants (*P* = 0.85), samples from participants with anti-S-RBD levels <300 U/mL and ≥300 U/mL (*P* = 0.40*)*, and samples from participants with or without symptoms of severe disease (fever and/or dyspnea) (*P* = 0.51*)*.

### Qualitative Ig responses and IFN_γ_ ELISpot responses

Among Ig seropositive participants, 71.3% (129/181) had a positive IFN_γ_ ELISpot, compared to 15.6% (14/90) of seronegative participants (*P* < 0.001, Table 1).

**Fig 2:**
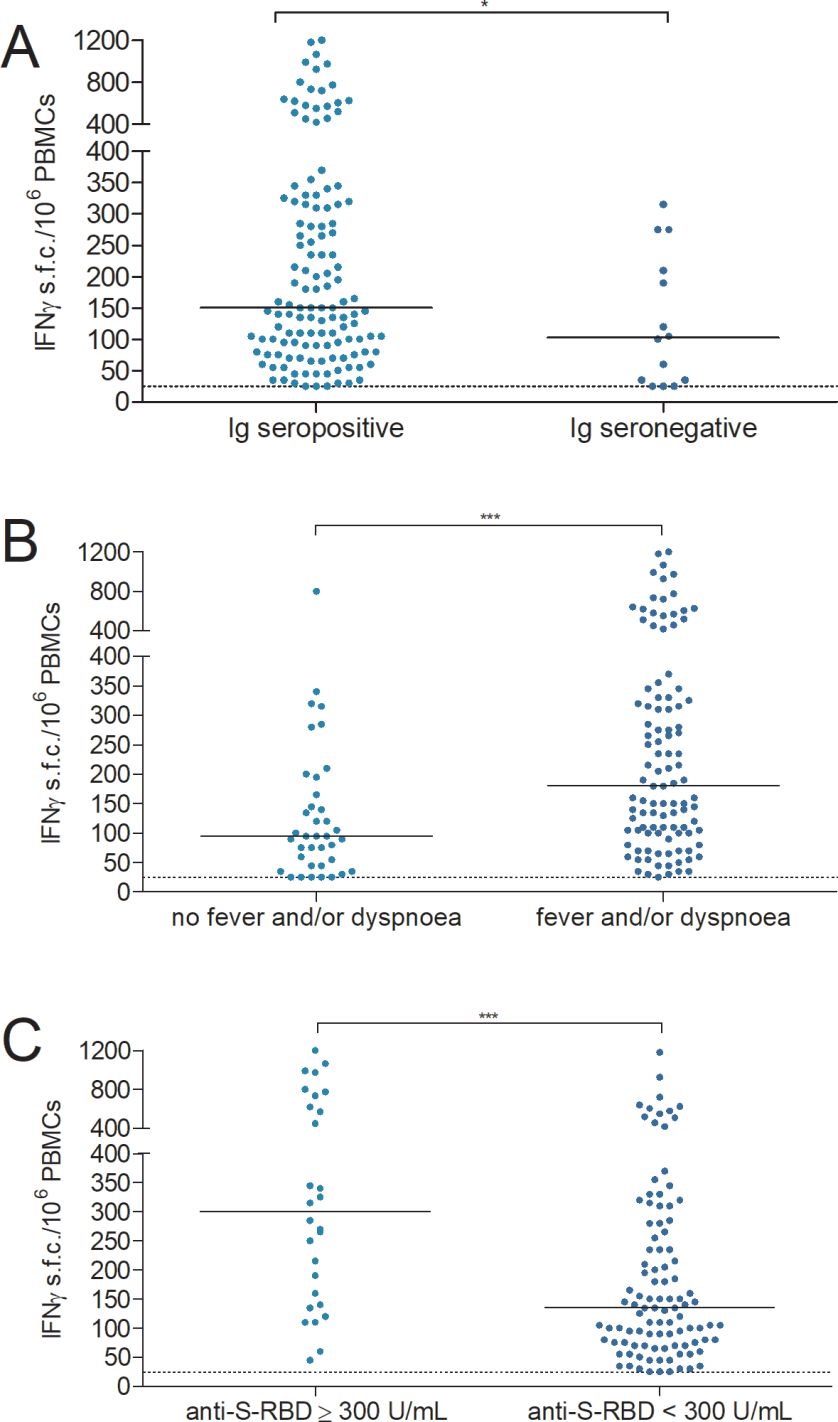
(**A**) Median positive IFN_γ_ ELISpot responses in Ig seropositive participants (*n* = 129) and Ig seronegative participants (*n* = 14). *P < 0.05. (**B**) Median positive IFN_γ_ ELISpot responses in participants without fever and/or dyspnea (*n* = 38) or with fever and/or dyspnea (*n* = 105). ***P < 0.001. (**C**) Median positive IFN_γ_ ELISpot responses in participants with anti-S-RBD levels >300 U/mL (*n* = 28) and participants with anti-S-RBD levels <300 U/mL (*n* = 101). ***P < 0.001. The dotted horizontal line shows the threshold for a positive IFN_γ_ ELISpot response.

The median response of a positive IFN_γ_ ELISpot for Ig seropositive participants [150 s.f.c./10^6^ PBMCs (IQR 85–318), *n* = 129] was higher than for Ig seronegative participants [103 s.f.c./10^6^ PBMCs (IQR 33–226), *n* = 14], *P* = 0.04 (Fig. 2A).

Participants with fever, anosmia, or aguesia were significantly more likely to have a positive IFN_γ_ ELISpot (Table 1). In Ig seropositive participants, age (*P* < 0.01), fever (*P* = 0.02), and ageusia (*P* = 0.02) were associated with a positive IFN_γ_ ELISpot (Table 2). In seronegative participants, no participant characteristics could be identified that were associated with a positive IFN_γ_ ELISpot (Table 2).

**TABLE 2:** ELISpot IFNγ response in Ig seropositive and seronegative participants (*n* = 271)

	Ig positive (*n* = 181)	ELISpot positive (*n* = 129)	ELISpot negative (*n* = 52)	Sig. (2-sided)	Ig negative (*n* = 90)	ELISpot positive (*n* = 14)	ELISpot negative (*n* = 76)	Sig. (2-sided)
Sex, *n* (%)								*P* = 1.00^*a*^
Male	58 (32.0)	44 (74.6)	15 (25.4)		29 (32.2)	4 (13.8)	25 (86.2)	
Female	123 (68.0)	85 (69.1)	38 (30.9)	*P* = 0.35	61 (67.8)	10 (16.4)	51 (83.6)	
Age (years), median (IQR)	47 (34–59)	49 (3–60)	43 (26–53)	*P* < 0.01^**^	41 (33–52)	39 (30–44)	43 (34–53)	*P* = 0.17
Fever, *n* (%)								*P* = 0.81
No	84 (46.4)	53 (63.1)	31 (36.9)		54 (60.0)	8 (14.8)	46 (85.2)	
Yes	97 (53.6)	76 (78.4)	21 (21.6)	*P* = 0.02^*^	36 (40.0)	6 (16.7)	30 (83.3)	
Anosmia, *n* (%)								
No	71 (39.2)	49 (69.0)	22 (31.0)		81 (90.0)	13 (16.0)	68 (84.0)	
Yes	110 (60.8)	80 (72.7)	30 (27.3)	*P* = 0.59	9 (10.0)	1 (11.1)	8 (88.9)	*P* = 1.00^*a*^
Ageusia, *n* (%)								*P* = 1.00^*a*^
No	61 (33.7)	37 (60.7)	24 (39.3)		80 (90.0)	13 (16.2)	67 (83.8)	
Yes	120 (66.3)	92 (76.7)	28 (23.3)	*P* = 0.02^*^	10 (10.0)	1 (10.0)	9 (90.0)	
Number of days between positive PCR and sample, median (IQR)	37 (24–212) (*n* = 33)	47 (25–222) (*n* = 21)	27 (21–111) (*n* = 11)	*P* = 0.22	29 (24–36)	29 (*n* = 1)	32 (24–37) (*n* = 10)	*P* = 0.91
Period of infection								*P* = 1.00^*a*^
6–9 months	150 (82.9)	108 (72.0)	42 (28.0)		60 (66.7)	10 (16.7)	50 (83.3)	
≤5 months	23 (12.7)	14 (60.9)	9 (39.1)	*P* = 0.22	22 (24.4)	3 (13.6)	19 (86.4)	
Missing^*b*^	8 (4.4)	7 (87.5)	1 (12.5)		8 (8.9)	1 (12.5)	7 (87.5)	

^*a*^ In case of expected counts <5, Fisher’s exact test was used.

^*b*^ Participants who could not be categorized in the first or second wave because of being asymptomatic or reporting non-specific symptoms. Significant values are displayed in bold. *p<0.05. **p<0.01.

### Quantitative IFN_γ_ responses and disease severity

Participants with fever and/or dyspnea had significantly higher IFN_γ_ responses [180 s.f.c./10^6^ PBMCs (IQR 100–330), *n* = 105] than participants without these symptoms [95 s.f.c./10^6^ PBMCs (IQR 45–173), *n* = 38], *P* < 0.001 (Fig. 2B).

### Quantitative anti-S-RBD responses and IFN_γ_ responses

Participants with anti-S-RBD levels ≥300 U/mL were significantly more likely to have a positive IFN_γ_ ELISpot (93.3%, 28/30) than those with anti-S-RBD levels <300 U/mL (66.9%, 101/151), *P* = 0.003 (Table 1). Apart from ageusia, no participant characteristics were identified as predictive of a positive IFN_γ_ ELISpot in participants with anti-S-RBD levels < 300 U/mL (Table S1).

Median positive IFN_γ_ responses in participants with anti-S-RBD levels ≥300 U/mL [300 s.f.c./10^6^ PBMCs (IQR 145–706), *n* = 28] were significantly higher than median positive IFN_γ_ responses in seropositive participants with anti-S-RBD levels <300 U/mL [135 s.f.c./10^6^ PBMCs (IQR 75–273), *n* = 101], *P* < 0.001 (Fig. 2C). Older age was associated with higher anti-S-RBD levels and higher IFN_γ_ responses; *r* (180) = 0.287, *P* < 0.001 and *r* (180) = 0.332, *P* < 0.001, respectively.

## DISCUSSION

The present study aimed to identify predictors of cellular immune responses in vaccine-naive residents of a southern province in the Netherlands during the first 8 months of the pandemic. Characteristic COVID-19 symptoms (i.e., anosmia and ageusia) and symptoms of more severe disease (i.e., fever) were more frequently reported in participants with a positive IFN_γ_ ELISpot response, while non-specific symptoms (i.e., cough, throat soreness, rhinorrhea, and headache) were associated with a negative IFN_γ_ ELISpot. A significant proportion of Ig seronegative participants (15.6%) showed a positive IFN_γ_ ELISpot response, in addition to the majority of seropositive participants who showed a positive IFN_γ_ ELISpot response.

In a large national collaboration evaluating the performance of SARS-CoV-2 immunoassays in individuals with mild infection, the Wantai SARS-CoV-2 Ab ELISA showed a sensitivity of 95.4% (95% confidence interval 92.8–97.1) when tested more than 14 days after symptom onset (16). Given the overall SARS-CoV-2 seroprevalence in our region after the first and second waves of 19.5%, approximately 1 out of the 90 seronegative cases could actually be false-negative (10). Another possible explanation for a cellular response in the absence of a humoral response could be that these participants were in the convalescent phase of their infection, as we only had PCR-based evidence of SARS-CoV-2 infection in one Ig seronegative participant with a positive IFN_γ_ ELISpot response. However, given the two waves of infection in the Netherlands in which the majority of participants may have been infected, these participants were most likely infected earlier, beyond the convalescent phase.

SARS-CoV-2-specific T-cell responses have been demonstrated in convalescents and close contacts of convalescents without detectable antibodies, suggesting that humoral seroprevalence may underestimate the true extent of the immune response to SARS-CoV-2 (3, 17, 18). Membrane-, nucleocapsid-, and non-structural proteins also induce cellular immune responses (3, 19). The use of a broad SARS-CoV-2 peptide pool covering the entire proteome of SARS-CoV-2, including membrane-, nucleocapsid-, envelope-, and non-structural protein-specific peptides in addition to S-specific peptides, may explain why a proportion of the Ig seronegative participants showed a positive IFN_γ_ ELISpot response. T-cell responses against SARS-CoV-2 epitopes have been demonstrated in truly unexposed individuals, possibly explained by cross-reactive T-cell memory responses against other members of the coronavirus family (19–21). In the present study, common clinical symptoms of COVID-19 such as fever and ageusia were associated with positive IFN_γ_ responses in seropositive individuals but not in seronegative individuals. Therefore, IFN_γ_ responses in seronegative participants might be explained by cross-reactive cellular responses. Studies of cross-reactive humoral immune responses between endemic human coronaviruses (HCoVs) and SARS-CoV-2 suggest that cross-reactive humoral responses do not protect against SARS-CoV-2 infection (22, 23). Whether cross-reactive cellular responses contribute to protection against contracting SARS-CoV-2 infection and severe COVID-19 remains to be elucidated. A recent study found higher frequencies of non-spike cross-reactive T-cells in household contacts who remained PCR-negative compared with those who developed a positive PCR, suggesting that these cross-reactive cellular responses may help prevent contracting SARS-CoV-2 infection (5). Another recent study described a better clinical outcome after SARS-CoV-2 infection in individuals recently infected with an endemic HCoV (24). It is therefore of interest to gain more insight into the factors that contribute to cellular responses, irrespective of whether this response is derived by an infection with SARS-CoV-2 or by other endemic HCoVs.

In the present study, we detected a SARS-CoV-2-specific IFN_γ_ memory response in 70.9% of seropositive participants. Previous studies have suggested the development of SARS-CoV-2-specific cellular immune responses in most hospitalized and non-hospitalized convalescents (2, 15). The median IFN_γ_ responses in our study were comparable to other studies focusing mainly on non-hospitalized individuals (3, 25). In line with previous studies, indicating higher IFN_γ_ responses in individuals with more severe disease, we found higher IFN_γ_ responses in participants with fever and/or dyspnea (3, 15, 21, 26). Early induction of cellular immune response protects against severe COVID-19 (1). Failure to induce early cellular responses might result in an increased viral load, resulting in tissue damage and a subsequent late hyper-inflammatory state with IFN_γ_ production (27).

Humoral antibody responses are dependent on CD4+ T-cell responses, as CD4+ cells enable B cells to produce high-affinity antibodies for isotype-switching and the generation of long-lasting memory responses (26). Previous studies have found a positive correlation between cellular responses and spike-specific humoral responses (15, 28–30). Our study showed that participants with anti-S-RBD responses ≥300 U/mL also had significantly higher SARS-CoV-2-specific IFN_γ_ responses than participants with <300 U/mL. Because of the observed correlations between humoral and cellular responses, in a population with relatively low antibody levels, possible less reactive cellular immune responses must be considered when estimating overall immune protection.

A strength of the present study is that we were able to include vaccine-naive participants with a wide spectrum of COVID-19. Thus, our study provides a model for estimating immune responses to natural SARS-CoV-2 infection in the general population. However, a limitation of our study is that, as the majority of participants were symptomatic, we could not thoroughly analyze cellular immune responses in completely asymptomatic individuals. Another limitation of this study is that for most participants onset of presumed infection was inferred from reported symptoms, and a PCR-proven infection was only available for 43 participants. However, for these participants, the period of infection inferred from reported symptoms correlated well with the period of PCR positivity. In the present study, only the Wuhan strain peptides were used to stimulate PBMCs. Although we did not perform additional experiments with newer variants of concern, recent reports have addressed this issue and demonstrated that cellular responses to the now dominant Omicron variant are largely conserved (7–9).

In conclusion, this study aimed to determine cellular immune responses in individuals with different humoral immune responses and different disease severities. We show that SARS-CoV-2-specific cellular immune responses are higher in individuals with higher humoral responses and in more severe diseases. Our study showed a cellular immune response in 15.6% of seronegative participants but to a lesser extent than in seropositive participants. Testing for both humoral and cellular immune responses may contribute to a more thorough assessment of the extent of immune responses to SARS-CoV-2 in a population. Future research is needed to determine what levels of SARS-CoV-2-specific cellular immune responses are required to protect against severe COVID-19.

## SUPPLEMENTAL MATERIAL

Supplemental material spectrum.00126-24-s0001.docx Table S1.

ASM does not own the copyrights to Supplemental Material that may be linked to, or accessed through, an article. The authors have granted ASM a non-exclusive, world-wide license to publish the Supplemental Material files. Please contact the corresponding author directly for reuse.
