# Supplementary material for: SARS-CoV-2 cellular and humoral responses in vaccine-naive individuals during the first two waves of COVID-19 infections in the southern region of The Netherlands: a cross-sectional population-based study
Source: Microbiol Spectr. 2024 Apr 30;12(6):e00126-24. doi: 10.1128/spectrum.00126-24 (PMC11237656; doi:10.1128/spectrum.00126-24)
Supplement: Supplemental material — Table S1. [file spectrum.00126-24-s0001.docx]

| **Supplementary Table 1: ELISpot IFNγ response in Ig seropositive participants with anti-S-RBD levels ≥300 U/mL or <300 U/mL (n=181)** | | | | | | | | | |
| --- | --- | --- | --- | --- | --- | --- | --- | --- | --- |
|  | **Anti-S-RBD ≥300 U/mL (n=30)** |  | **ELISpot positive**  **(n=28)** | **ELISpot negative**  **(n=2)** | **Sig. (2-sided)** | **Anti-S-RBD <300 U/mL (n=151)** | **ELISpot positive**  **(n=101)** | **ELISpot negative**  **(n=50)** | **Sig. (2-sided)** |
| **Sex**, n (%)  Male  Female | 9 (30.0)  21 (70.0) |  | 9 (100)  19 (90.5) | 0 (0)  2 (9.5) | *p*=1.00^a^ | 49 (32.5)  102 (67.5) | 35 (71.4)  66 (64.7) | 14 (28.6)  36 (35.3) | *p*=0.41 |
| **Age** (years) median, (IQR) | 61 (50-65) |  | 61 (49-66) | - | *p*=0.46 | 44 (32-57) | 44 (34-59) | 43 (26-53) | ***p=*0.03*** |
| **Fever**, n (%)  No  Yes | 10 (33.3)  20 (66.7) |  | 9 (90.0)  19 (95.0) | 1 (10.0)  1 (5.0) | *p*=1.00^a^ | 74 (49.0)  77 (51.0) | 44 (59.5)  57 (74.0) | 30 (50.5)  20 (26.0) | *p*=0.06 |
| **Anosmia**, n (%)  No  Yes | 14 (46.7)  16 (53.3) |  | 13 (92.9)  15 (93.8) | 1 (7.1)  1 (6.2) | *p*=1.00^a^ | 57 (37.7)  94 (62.3) | 36 (63.2)  65 (69.1) | 21 (36.8)  29 (30.9) | *p*=0.45 |
| **Ageusia**, n (%)  No  Yes | 9 (30.0)  21 (70.0) |  | 8 (88.9)  20 (95.2) | 1 (11.1)  1 (4.8) | *p*=0.52 | 52 (34.4)  99 (65.6) | 29 (55.8)  72 (72.7) | 23 (44.2)  27 (27.3) | ***p*=0.04*** |
| **Number of days between positive PCR and sample**, median (IQR) | 222 (75-253) (n=4) |  | 222 (75-253) (n=4) | - |  | 36 (22-161) (n=28) | 44 (22-212) (n=17) | 35 (21-111) (n=11) | *p*=0.43 |
| **Period of infection**  6-9 months  ≤5 months  Missing^b^ | 28 (93.3)  1 (3.3)  1 (3.3) |  | 26 (92.9)  1 (100)  1 (100) | 2 (7.1)  0 (0)  0 (0) | *p*=1.00^a^ | 122 (80.8)  22 (14.6)  7 (4.6) | 82 (67.2)  13 (59.1)  6 (85.7) | 40 (32.8)  9 (40.9)  1 (14.3) | *p*=0.46 |
| ^a^In case of expected counts less than 5, Fisher’s Exact Test was used. ^b^Participants who could not be categorised in the first or second wave because of being asymptomatic or reporting non-specific symptoms. **p*<0.05. | | | | | | | | | |
|  |  |  |  |  |  |  |  |  |  |
